# Live fast, die young? A review on the developmental trajectories of ADHD across the lifespan

**DOI:** 10.1016/j.euroneuro.2018.08.001

**Published:** 2018-10

**Authors:** Barbara Franke, Giorgia Michelini, Philip Asherson, Tobias Banaschewski, Andrea Bilbow, Jan K. Buitelaar, Bru Cormand, Stephen V. Faraone, Ylva Ginsberg, Jan Haavik, Jonna Kuntsi, Henrik Larsson, Klaus-Peter Lesch, J. Antoni Ramos-Quiroga, János M. Réthelyi, Marta Ribases, Andreas Reif

**Affiliations:** a Department of Human Genetics, Donders Institute for Brain, Cognition and Behaviour, Radboud University Medical Center, Nijmegen, The Netherlands; b Department of Psychiatry, Donders Institute for Brain, Cognition and Behaviour, Radboud University Medical Center, Nijmegen, The Netherlands; c King's College London, Institute of Psychiatry, Psychology & Neuroscience, Social, Genetic & Developmental Psychiatry Centre, London, UK; d Department of Child and Adolescent Psychiatry and Psychotherapy, Central Institute of Mental Health, Medical Faculty Mannheim, University of Heidelberg, Mannheim, Germany; e Attention Deficit Disorder Information and Support Service (ADDISS), Edgware, UK; f ADHD-Europe, Brussels, Belgium; g Radboud University Medical Center, Donders Institute for Brain, Cognition and Behaviour, Department of Cognitive Neuroscience, Nijmegen, The Netherlands; h Department of Genetics, Microbiology and Statistics, Faculty of Biology, Universitat de Barcelona, Barcelona, Catalonia, Spain; i Centro de Investigación Biomédica en Red de Enfermedades Raras (CIBERER), Instituto de Salud Carlos III, Spain; j Institut de Biomedicina de la Universitat de Barcelona (IBUB), Barcelona, Catalonia, Spain; k Institut de Recerca Sant Joan de Déu (IR-SJD), Esplugues de Llobregat, Catalonia, Spain; l Departments of Psychiatry and of Neuroscience and Physiology, State University of New York Upstate Medical University, New York, USA; m K.G. Jebsen Centre for Neuropsychiatric Disorders, Department of Biomedicine, University of Bergen, Bergen, Norway; n Department of Medical Epidemiology and Biostatistics, Karolinska Institutet, Stockholm, Sweden; o Department of Clinical Neuroscience, Centre for Psychiatry Research, Karolinska Institutet, Stockholm, Sweden; p Division of Psychiatry, Haukeland University Hospital, Bergen, Norway; q Division of Molecular Psychiatry, Center of Mental Health, University of Würzburg, Würzburg, Germany; r Laboratory of Psychiatric Neurobiology, Institute of Molecular Medicine, I.M. Sechenov First Moscow State Medical University, Moscow, Russia; s Department of Translational Neuroscience, School for Mental Health and Neuroscience (MHeNS), Maastricht University, Maastricht, The Netherlands; t Department of Psychiatry, Hospital Universitari Vall d'Hebron, Barcelona, Catalonia, Spain; u Psychiatric Genetics Unit, Vall d'Hebron Research Institute (VHIR), Barcelona, Catalonia, Spain; v Biomedical Network Research Centre on Mental Health (CIBERSAM), Barcelona, Catalonia, Spain; w Department of Psychiatry and Legal Medicine, Universitat Autònoma de Barcelona, Barcelona, Catalonia, Spain; x Department of Psychiatry and Psychotherapy, Semmelweis University, Budapest, Hungary; y MTA-SE NAP-B Molecular Psychiatry Research Group, Hungarian Academy of Sciences, Budapest, Hungary; z Department of Psychiatry, Psychosomatic Medicine and Psychotherapy, University Hospital Frankfurt, Frankfurt am Main, Germany

**Keywords:** Developmental trajectory, Treatment, Comorbidity, Cognitive impairment, Genetics, Adult-onset ADHD

## Abstract

Attention-deficit/hyperactivity disorder (ADHD) is highly heritable and the most common neurodevelopmental disorder in childhood. In recent decades, it has been appreciated that in a substantial number of cases the disorder does not remit in puberty, but persists into adulthood. Both in childhood and adulthood, ADHD is characterised by substantial comorbidity including substance use, depression, anxiety, and accidents. However, course and symptoms of the disorder and the comorbidities may fluctuate and change over time, and even age of onset in childhood has recently been questioned. Available evidence to date is poor and largely inconsistent with regard to the predictors of persistence versus remittance. Likewise, the development of comorbid disorders cannot be foreseen early on, hampering preventive measures. These facts call for a lifespan perspective on ADHD from childhood to old age. In this selective review, we summarise current knowledge of the long-term course of ADHD, with an emphasis on clinical symptom and cognitive trajectories, treatment effects over the lifespan, and the development of comorbidities. Also, we summarise current knowledge and important unresolved issues on biological factors underlying different ADHD trajectories. We conclude that a severe lack of knowledge on lifespan aspects in ADHD still exists for nearly every aspect reviewed. We encourage large-scale research efforts to overcome those knowledge gaps through appropriately granular longitudinal studies.

## 1 Introduction

Attention-deficit/hyperactivity disorder (ADHD) is a neurodevelopmental condition that typically starts during childhood or early adolescence and is thought to follow a trait-like course. The clinical disorder is defined by age-inappropriate levels of inattention and/or hyperactivity-impulsivity interfering with normal development, or functioning, of a person. Although ADHD carries the stigma of being a consequence of modern lifestyle, the first mentioning of the syndrome dates back to the late 18th century (Faraone et al., 2015). Historically, ADHD was described mainly in school-age boys (Still, 2006). Later, it was recognised that many girls have similar problems – yet often remain unrecognised and, consequently, undiagnosed. During the past decades, it has been demonstrated that ADHD is common in all countries studied (Fayyad et al., 2017, Polanczyk et al., 2014), and that it seriously affects the productivity, life expectancy, and quality of life throughout the lifespan of patients (Erskine et al., 2013). Importantly, it took until the late 20th century before it could convincingly be shown that ADHD also exists in adults, and that continuity exists from childhood to adulthood (Wood et al., 1976) calling for a lifespan perspective on the disorder, embracing clinical course and presentation as well as according research on the underlying neurobiology.

As discussed in detail in this review, the clinical presentation of ADHD is very heterogeneous, with a wide spectrum of severity and symptoms that partially overlap with other conditions. In fact, ADHD symptoms can be observed transiently not only in psychiatric disorders, but also in somatic diseases and physiological states, such as after sleep deprivation or during over-exhaustion (Poirier et al., 2016). This complex clinical picture has led to a need to define core diagnostic attributes of ADHD, such as age of onset, continuity of symptoms and their appearance under various circumstances, symptom counts, and exclusion criteria. Although the diagnostic criteria have been revised multiple times, the core clinical description of ADHD has remained essentially unchanged during several decades. In the latest version of the Diagnostic and Statistical Manual of Mental Disorders (DSM-5), it is simply stated that “ADHD begins in childhood” and that it often manifests itself in pre-school age (APA, 2013). This apparent simplification does not imply that every person with ADHD will have an identical clinical picture, or that impairment is linearly dependent on symptom counts or age of onset.

Unfortunately, the fields of research on childhood and adulthood ADHD have operated in relative isolation, mainly due to a historical hiatus between child/adolescent and adult psychiatry. However, the need for a lifespan perspective is becoming increasingly apparent, and is enhanced by the voices of patients and their representatives (see e.g. the Textbox below). In this review, we present selected literature to summarise the current knowledge on ADHD from a developmental, lifespan perspective. Preventive measures as well as age-specific diagnostics and interventions require knowledge about the highly dynamic changes in ADHD presentation from childhood to adulthood, and our review of the current state of knowledge is intended to provide researchers and clinicians with an overview on the trajectory of this disorder. We focus on phenotypic changes across the lifespan, age of onset issues (also covering the recent discussion on adult onset ADHD), lifespan aspects of comorbidity, pharmacological and non-pharmacological treatment, as well as aspects of disease outcome with and without treatment. In addition, we summarise knowledge on cognitive and neuroimaging changes during life with the disorder and touch on lifespan aspects in the study of genetic and environmental risk factors for the disorder as well as the interplay of those two. Subsequent to these reviews, we point out existing knowledge gaps and identify needs for further research.

Textbox **The patient perspective – contributed by Andrea Bilbow, President of ADHD-Europe and Founder and Chief Executive of the Attention Deficit Disorder Information and Support Service (ADDISS)**

Despite considerable advances in our understanding of ADHD, patients still experience significant problems gaining access to the support and treatment they need. Service user groups are established in most European countries. One of these groups, ADHD-Europe (http://www.adhdeurope.eu), aims to advance the rights of, and advocate on every level throughout Europe, for people affected by ADHD and comorbid conditions, helping them reach their full potential. ADHD-Europe plays a critical role in promoting awareness of ADHD and evidence-based treatments, facilitate the efforts of national and regional ADHD support groups, and advocate to European Institutions for the delivery of appropriate services for children, adolescents, and adults with ADHD.

Currently, the experience of service users is that they are often directed towards services that do not recognise the specific problems related to ADHD. This is a particular problem for adults with ADHD, although the quality and availability of child services also varies considerably across different countries and regions. Patients often feel there is nowhere to go for help with ADHD-related problems. In most cases, adult social care does not yet cater for ADHD. At the same time, old age, learning disability, and mental health teams often do not consider ADHD as a mental health problem, which then falls between the cracks, with no service provision available. The lack of support contributes to people with ADHD experiencing more severe problems with age. When faced with challenges, they may feel overwhelmed and develop or exacerbate comorbid problems such as anxiety, depression, and drug use, which then complicate ADHD. Much more work around social care and support is required.

Even where there are health-care services for ADHD, the approach is often to use drugs alone without providing the additional psychosocial support that is required. In most cases, it is not enough to just see someone and give a prescription. Health-care professionals need to consider the environmental circumstances of each individual, and clinicians need to think “what more can I do for that person”. Practical support can come in many ways, but the key is to have a single person coordinating the treatment package – one who is aware of the individual circumstances and difficulties faced by people being treated for ADHD and can provide practical support as required.

Medication is viewed as an essential tool to ensure that other supports become effective. However, medication often does not fully control all of the symptoms of ADHD, and most importantly, does not build skills. Organisational skills can improve with medication – being less distracted and more able to stay on task and get things done – but people still need daily life skills. For example, someone may remain overwhelmed by tasks such as how to tidy up a room, how or organise and complete paperwork, and how to plan ahead for the successful completion of tasks and ideas.

Scaffolding and support for people with ADHD is complex and specialised. One of the problems is that support is often offered for short periods, such as 3 months, but then they are on your own. Lifelong scaffolding and support may be required. To fill the gap in service provision, a considerable amount of necessary practical and psychosocial support is currently provided by the voluntary sector. One approach advocated by ADHD-Europe is to invest in the voluntary sector to provide the support services required, rather than rely on good-will of patient support groups. This is particularly important as the role of the voluntary sector is limited in what they can do due to lack of funding.

## 2 Phenotype of ADHD across the lifespan – course and changes in presentation over time

ADHD is defined as a persistent, trans-situational pattern of inattention and/or hyperactivity-impulsivity that is inappropriate to the developmental stage and interferes with functioning or development (APA, 2013). Importantly, ADHD symptoms as such do not reflect a manifestation of oppositional behaviour, defiance, hostility, or failure to understand tasks or instructions, although such problems are often seen to accompany ADHD. Meta-analysis of longitudinal follow-up studies of children with ADHD suggests that at least 15% continue to meet full diagnostic criteria for ADHD by the age of 25 years, and a further 50% meet criteria for ADHD in partial remission, with persistence of subthreshold symptoms still causing impairment (Faraone et al., 2006). However, there is considerable heterogeneity in those estimates, as e.g. more recent estimates of persistence into young adulthood for children and adolescents diagnosed with DSM-IV combined type ADHD in Europe are much higher (up to 80%) (Cheung et al., 2015a, van Lieshout et al., 2016b), perhaps reflecting the severity of cases included in these studies, and/or the use of informant- rather than self-ratings.

Prevalence rates for ADHD in children range around 6.5% (Polanczyk et al., 2007). Estimates for adults vary widely across studies, but average around 2.5–3.4% in meta-analysis (Fayyad et al., 2007, Simon et al., 2009). Fayyad et al. (2017) cite estimates ranging between 1.4 and 3.6%. Such variation is almost certainly due to methodological differences in the way the diagnostic criteria are applied, including the childhood onset of symptoms, the methods to capture the 18 behavioural symptoms used to define the condition, and the application of impairment criteria (Willcutt, 2012). Definitions of impairment are a particular issue, because ADHD symptoms are known to be continually distributed throughout the population, with no clear separation between those with and without ADHD (Mulligan et al., 2008). The disorder is therefore defined by high levels of symptoms when they interfere with or reduce the quality of social, academic, or occupational functioning (NICE, 2013). This might also at least in part underlie the discrepancy between cross-sectional, epidemiological studies showing adult ADHD prevalence rates almost as high as childhood prevalence rates (Fayyad et al., 2017), and longitudinal samples that suggest adult prevalence rates to be much lower (Faraone et al., 2015).

Characteristic changes occur in the profile of ADHD symptoms throughout development. Very young children are more likely to display externalising symptoms such as hyperactive-impulsive behaviour, while in middle childhood inattentive symptoms become more apparent, and by late adolescence and in adulthood it is inattention that tends to persist, while there is a decline in the more objective signs of (motor) hyperactivity (Francx et al., 2015b, Willcutt et al., 2012). Emotional lability, however, becomes a growing burden, which can even dominate the clinical picture. It is this changing profile and instability in the balance of symptoms presenting throughout development that led to disbanding of the DSM-IV ADHD subtypes of predominantly inattentive, predominantly hyperactive-impulsive, and combined subtypes; those are now referred to as clinical presentations in DSM-5. Although many adults present with predominantly inattentive symptoms, this is not to mean they have the same rate of hyperactive or impulsive symptoms compared to age-matched controls. Persistence of more overt hyperactivity-impulsivity is seen at higher rates especially among those with some of the most severe comorbid problems related to ADHD, such as substance abuse and antisocial behaviour (Huntley et al., 2012).

Sex differences in the rates of ADHD are prominent but change throughout development in both clinical and community settings (Kooij et al., 2010, Larsson et al., 2011). Typically, in child and adolescent clinics, around 80% of ADHD cases are male, whereas in adult clinics, the proportion of males is closer to 50% (Kooij et al., 2010). One possible reason for the predominance of males in child clinics is the greater hyperactivity-impulsivity levels they show compared to girls, who are more likely to display predominantly inattentive symptoms and less overt disruptive behaviours. Several lines of evidence support this notion. For example, an epidemiological survey of childhood ADHD found the ratio of male to female cases was 7.0 for the hyperactive-impulsive subtype, 4.9 for the combined subtype, and 3.0 for the inattentive subtype of ADHD. However, there was still a greater percentage of boys with ADHD of any subtype (3.6%) compared to girls (0.85%). This is consistent with mean scores for ADHD symptoms in general population samples that show that, as a group, boys have higher levels of both inattentive and hyperactive-impulsive symptoms than girls. Interestingly, by late adolescence, while sex differences in inattention appear to remain, the level of hyperactivity-impulsivity in boys declines to the level of the girls (Larsson et al., 2011), suggesting that the expression of core ADHD symptoms is more similar across the sexes in the adult population. In addition to sex differences in the expression of core ADHD symptoms, it is likely that comorbid problems contribute to different referral rates for boys compared to girls, with boys presenting with more externalising disruptive behaviours and learning problems. In adults, it is well known that women are more likely to seek help for mental health problems, impacting on referral rates, but levels of comorbid disorders appear to be similar in both men and women with ADHD (Biederman et al., 2004).

While diagnostic criteria for ADHD originate from diagnosis of children, the currently used diagnostic criteria, when applied to adults, are also well-validated and therefore appear to work well in the classification of clinical cases of the disorder (Asherson et al., 2010). Validation of the diagnostic criteria also requires the prediction of functional and clinical impairments (NICE, 2018). Yet, most clinical experts are aware of a broader expression of symptoms commonly reported by adults with ADHD (Asherson et al., 2016, Kooij et al., 2010) falling into three main categories: age-adjusted expression of core ADHD symptoms, behaviours reflecting problems with self-regulation (executive functions), and additional problems that are commonly seen in ADHD. Age-adjusted ADHD core symptoms include some items in DSM-5 (APA, 2013) and e.g. internal restlessness, ceaseless unfocused mental activity, and a difficulty focusing on conversations. Problems with self-regulation are e.g. problems with controlling impulses, switching attention, regulating emotional responses, initiating tasks, and problem-solving. Interestingly, while these are strongly correlated with the core ADHD symptoms of inattention and hyperactivity-impulsivity, they do not correlate strongly with neuropsychological tests of executive or top-down cortical control (Barkley, 2010, Barkley and Fischer, 2011), suggesting that, like core ADHD symptoms, they result from deficits across multiple neural networks and cognitive processes. The additional problems seen in many adults include sleep problems and low self-esteem (Kooij et al., 2010). Furthermore, a study that carefully selected adult ADHD cases with no evidence for a comorbid disorder nevertheless found high rates of general mental health symptoms, such as subthreshold anxiety and depression (Skirrow and Asherson, 2013).

## 3 Age of onset and current discussion on adult onset of ADHD

**Fig. 1:**
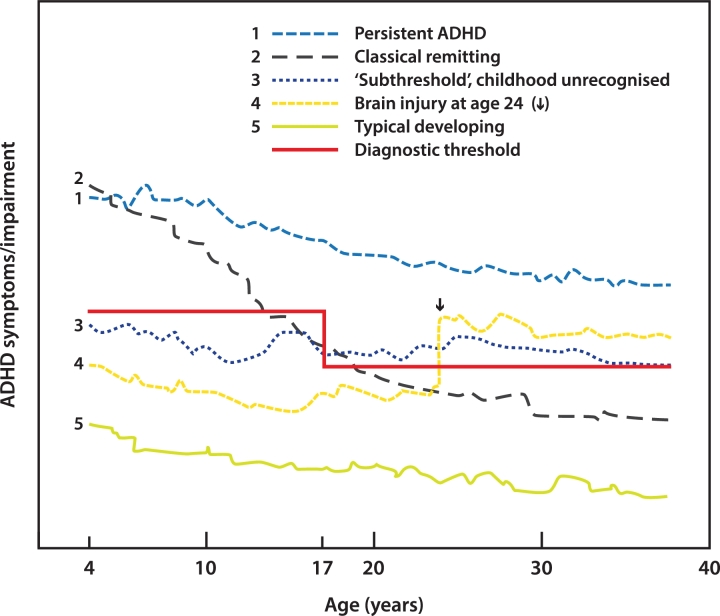
Theoretical developmental trajectories of ADHD across the lifespan. Details are given in the text.

As pointed out above, ADHD has always been viewed as a childhood-onset condition, although the first age at onset criterion was not seen until the advent of DSM-III, which required onset prior to age 7 years (APA, 1980). This threshold was raised to age 12 years in DSM-5 (APA, 2013). Patients, however, can meet symptom and impairment criteria at later ages. Sometimes, such cases are clearly due to brain injuries (Fig. 1). If so, they are usually classified as “secondary” or “acquired” ADHD, to be distinguished from the childhood onset of ADHD discussed above (Schachar et al., 2015). Longitudinal studies report a two-fold relative risk of receiving a diagnosis of ADHD after mild traumatic brain injury (TBI) (Adeyemo et al., 2014); more severe brain trauma carries an even higher risk for ADHD (Schachar et al., 2015). Overall, 15–50% of children with TBI develop secondary ADHD (Schachar et al., 2015), which may be clinically indistinguishable from idiopathic ADHD. Because people with ADHD are at risk for accidents (Dalsgaard et al., 2015b), ADHD may be a risk factor for head injuries (Fann et al., 2002), although this has been difficult to establish (Adeyemo et al., 2014). Nevertheless, it is possible that some patients with ADHD emerging subsequent to TBI had undiagnosed ADHD or subthreshold ADHD prior to their injury.

In contrast to the well-established link between TBI and ADHD in adulthood, the idea that idiopathic ADHD arises, *de novo*, in adulthood is controversial. Three population studies estimated high rates of adult-onset idiopathic ADHD, with a prevalence of 2.7% in New Zealand (NZ) (Moffitt et al., 2015), 10.3% in Brazil (Caye et al., 2016), and 5.5% in the United Kingdom (UK) (Agnew-Blais et al., 2016). The authors concluded that ADHD can onset in adulthood and that the adult-onset form of the disorder is categorically distinct from the childhood-onset form. Yet these conclusions were premature. All three studies had some serious limitations (Faraone and Biederman, 2016). Firstly, the age of the adults in the studies from Brazil and the UK was only 18 to 19 years, so these studies provide no information about most of the adult period. Secondly, in all three studies, the rates of childhood-onset adult ADHD were much lower than expected (Fayyad et al., 2017), suggesting that many of the childhood-onset cases may have been missed and misdiagnosed as adult onset. Thirdly, all three studies suffer from the “false positive paradox”, which is the mathematical fact that, in the absence of perfect diagnostic accuracy, many of the diagnoses in a population study will be false positives. For example, if the prevalence of adult ADHD is 5%, and the false positive rate is only 5%, then half of the adult ADHD diagnoses in a population study will be false positives. Consistent with this idea, Sibley et al. (2017) concluded that adult onset of ADHD is rare, and that most people exceeding the symptom threshold for diagnosis are, on closer examination, false positives. A fourth caveat for the three studies is that the distinction between childhood onset and adulthood onset was confounded by the method of diagnosis: the former diagnoses were based on parent-report, whereas the latter were based on self-report. This is a problem, because another longitudinal study found that current symptoms of ADHD were under-reported by adults who had ADHD in childhood and over-reported by adults who did not have ADHD in childhood (Sibley et al., 2012). Moreover, considerable evidence suggests that, compared with informant-reports, self-reports of ADHD in adults are less reliable. The noise added by self-reports can be seen in the very low heritability for adult ADHD reported by the UK study (35%; Agnew-Blais et al., 2016), which contrasts with the higher heritability of adult ADHD from parent report or using diagnostic criteria (Brikell et al., 2015).

In all three studies, a participant was defined as having “adult-onset” ADHD only if full diagnostic criteria for ADHD had not been achieved at prior assessments. In each study, however, many of the “adult-onset” cases had evidence of psychopathology in childhood. In the NZ study, in their childhood years, the adult-onset ADHD group had more teacher-rated symptoms of ADHD, were more likely to have been diagnosed with conduct disorder (CD), and were more likely to have had a combined parent-teacher report of ADHD symptom onset prior to age 12 years (Moffitt et al., 2015). In the UK study, the adult-onset cases had significantly elevated rates of ADHD symptoms, CD, and oppositional defiant disorder (ODD) in childhood (Agnew-Blais et al., 2016). In the study from Brazil, only about a third of the adult-onset cases were free of ADHD and CD symptomatology in childhood (Caye et al., 2016). These population studies mirror retrospective reports from referred cases, in which many late adolescent and adult onset cases of ADHD had childhood histories of psychopathology (Chandra et al., 2016).

Faraone and Biederman (2016) suggested that apparent cases of adult-onset ADHD are mostly due to the existence of subthreshold childhood ADHD. For example, a prospective population study defined “subthreshold ADHD” (Fig. 1) as having three or more inattentive symptoms or three or more hyperactive-impulsive symptoms (Lecendreux et al., 2015). It found that new-onset cases of ADHD in adolescence were significantly more likely to have had subthreshold ADHD at baseline. In subthreshold cases, the onset of symptoms and impairment could be separated by many years, particularly among those with supportive internal resources (e.g., high intelligence) or supportive social environments.

Given the issues discussed above, and until more evidence is available, it seems best to refer to the adult-onset cases reported by the studies from NZ, Brazil, and the UK as *apparent* adult-onset ADHD (AAOA). An urgent clinical question is whether stimulant treatment is appropriate for AAOA. This issue has not yet been systematically assessed. From a clinical perspective, AAOA cases require extra caution. Although many show significant functional impairments that require treatments for ADHD, some may have other disorders.

In conclusion, substantial research indicates that a diagnosable ADHD syndrome can arise in adulthood subsequent to brain injury. Other forms of apparent adult-onset ADHD may exist, but many of these are likely to have had undiagnosed ADHD or subthreshold ADHD in youth.

## 4 Comorbidity profile changes over time

To complicate matters further, not only does the clinical phenotype of ADHD change over the lifespan, but comorbid conditions might dominate the initial appearance of a patient. This is of high relevance as ADHD patients frequently suffer from psychiatric and non-psychiatric comorbid conditions, posing significant clinical and public health problems (Angold et al., 1999). Throughout the lifespan, the specific pattern of comorbidities changes substantially (Costello et al., 2003, Taurines et al., 2010): in short, while in children oppositional defiant disorder (ODD) and conduct disorder (CD) are the most prevalent comorbid conditions, substance use disorders (SUDs) become more and more of a problem during adolescence and even more so in adulthood. The comorbidity pattern of adult ADHD is highly diverse, and in addition to SUDs encompasses mood and anxiety disorders, antisocial personality disorder (ASP), sleep disorders (Jacob et al., 2007), as well as many somatic diseases (Instanes et al., 2016). The developmental trajectory, risk factors, and moderators of this lifelong comorbidity course, however, are currently only poorly understood and require future longitudinal studies. Below, information on the most prominent ADHD comorbidities is given.

### 4.1 Autism spectrum disorders, tics, and learning disorders

ADHD and symptoms of autistic spectrum disorders (ASDs) often co-exist, as 20–50% of children with ADHD also meet criteria for ASDs (Rommelse et al., 2011). Several studies have shown social deficits, peer relationship, and empathy problems to be common in ADHD, and accordingly, the DSM-5 finally allows a comorbid diagnosis of ADHD and ASD. The ADHD-ASD comorbidity has mainly been studied in children; a review and meta-analysis, however, support its existence also in adults (Hartman et al., 2016). Recent data from a large, register-based study from Sweden suggest that the comorbidity has its roots in shared genetic/familial factors (Ghirardi et al., 2017). Tic disorders occur in up to 3–4% of the population (Robertson et al., 2009, Roessner et al., 2011) and are seen in 10 to 20% of children with ADHD (Cohen et al., 2013, Steinhausen et al., 2006). Over the course of years, tic severity typically peaks between 8 and 12 years of age. The natural history of tics usually shows a marked decline during adolescence (Cohen et al., 2013). Population studies suggest that intellectual disability may be more common (up to 5–10 times) in ADHD than in children without ADHD (Simonoff et al., 2007). About 25–40% of all patients with ADHD have major reading and writing difficulties, and many show co-existing language disorders (Sciberras et al., 2014, Willcutt et al., 2012). Similarly, there is a considerable overlap between ADHD and disorders of arithmetical skills (Hart et al., 2010, Rapport et al., 1999). As for ASD, hardly any information is yet available on the lifespan trajectories of ADHD with such comorbidities.

### 4.2 Rule-breaking behaviours

The comorbidity of ADHD with antisocial behaviours is of particular societal relevance, as ADHD seems to convey an increased risk for violence and incarceration especially in the context of such comorbidity (Rosler et al., 2004). In children and adolescents, both clinical and epidemiological studies show a high prevalence of comorbidity of ADHD with ODD/CD, ranging from 25% up to 80% (median odds ratio (OR): 10) in different studies. ODD and CD predict a more severe clinical symptomatology, more severe functional impairments, higher persistence of ADHD into adulthood, and worse outcome of the disorder. They also mediate the risk for the development of other problems, such as substance use and depression (Burke et al., 2002, Hill, 2002). Comorbid ODD in childhood seems to also increase the risk for CD/ASP and depression in ADHD later in life, and comorbid CD does even more (Biederman et al., 2008, Mannuzza et al., 2004). Increased impulsivity may be an important risk factor within the ADHD group for these negative outcomes (Retz and Rosler, 2009, Storebo and Simonsen, 2016). However, ADHD-affected children without these comorbidities may also develop antisocial behaviours later in life (Jensen et al., 1997, Loeber et al., 1995, Mannuzza et al., 2004). SUDs and environmental variables might be important moderators in this, although this has yet to be formally established.

### 4.3 Substance use disorders

Another (related) issue of importance in ADHD across the lifespan is the liability to develop addictions. The earlier onset and increased use of tobacco, alcohol, and illicit substances in adolescents with ADHD compared to controls has been demonstrated in various studies, and a high prevalence of drug abuse or dependency (9–40%) is also reported in adulthood (Buchmann et al., 2009, Jacob et al., 2007, Mannuzza et al., 1993, Milberger et al., 1997). A meta-analysis of cohort studies confirmed that childhood ADHD significantly increases the risk for nicotine use in middle adolescence (OR: 2.36, 1.71–3.27) and the risk for alcohol use disorder during young adulthood (OR: 1.35, CI: 1.11–1.64) (Charach et al., 2011). This meta-analysis also suggested that children with ADHD may have an elevated risk for cannabis use and psychoactive substance use as young adults, but significant heterogeneity exists between studies, and the association with drug use disorder was highly influenced by a single study. As another meta-analysis showed, controlling for comorbid disorders (particularly CD) substantially weakened the association between ADHD and SUDs; in fact, it could not be confirmed that ADHD increases the risk for SUDs beyond the effects of CD/ODD (Serra-Pinheiro et al., 2013). Looked at from the other side, in adult patients suffering from alcohol abuse, 30–70% suffered from childhood ADHD, and 15–25% still displayed the disorder as adults.

### 4.4 Mood and anxiety disorders

Adult ADHD is significantly comorbid with anxiety disorders (up to 25%; median OR: 3.0) and major depression (5–20%; median OR: 5.5). Although these disorders are among the most common comorbidities of ADHD, especially in adolescents (Meinzer et al., 2013) and adults (Jacob et al., 2007), surprisingly little is known about the developmental trajectories of such comorbidity. As mentioned above, ODD/CD seem to be associated with later-life affective disorders; also, depressive and anxious symptoms during childhood and adolescence go along with increased risk for adult-life depression (as do general risk factors for depression), although often no such antecedents can be found in adult ADHD with depression. Rather, childhood ADHD itself seems to be a risk factor for the later development of depression, which can be reduced by e.g. methylphenidate treatment (Chang et al., 2016).

### 4.5 Disruptive mood dysregulation disorder and bipolar disorder

An issue of increasing interest in research and clinic is the presence of extreme and uncontrolled emotional and mood changes in both children and adults with ADHD. Indeed, in DSM-5, problems with emotion regulation are listed as characteristic features of ADHD that support the diagnosis. Emotional dysregulation and ADHD are known to share genetic risks (Merwood et al., 2014). In adult ADHD, emotional dysregulation occurs also in the absence of comorbid disorders (Skirrow and Asherson, 2013), is an independent predictor of impairment (Barkley and Fischer, 2010, Skirrow and Asherson, 2013), and responds to both stimulants and atomoxetine (Moukhtarian et al., 2017). In some cases, specialist referral is advised to ensure accurate diagnosis, because the problems may be complex and predictive of particular adverse outcomes (Sobanski et al., 2010, Stringaris and Goodman, 2009). Comorbidity with bipolar disorder and borderline personality disorder both need to be considered in adults, since ADHD may co-exist with these conditions, but may also mimic them in the presence of severe emotional dysregulation (Asherson et al., 2016). Cross-sectional epidemiological studies as well as family-based studies show that there is increased comorbidity between bipolar disorder and current or lifetime ADHD; mutual comorbidity rates are around 20% (Kessler et al., 2006). The developmental trajectories of this comorbidity are unclear, however, since bipolar disorder is rare in pre-adolescents, even when severe irritability and anger are prominent in this group (Brotman et al., 2006). The new DSM-5 diagnosis “disruptive mood dysregulation disorder (DMDD)” for children up to age 8 years exhibiting persistent irritability, intolerance to frustration, and frequent episodes of extreme behavioural dyscontrol captures those symptoms, yet the course of this syndrome into full-blown bipolar disorder is far from established. Importantly, most of the DMDD patients also meet criteria for ADHD.

## 5 Treatment and response to treatment over time – pharmacological and non-pharmacological treatments

The continuity of treatment of ADHD across the lifespan is an issue that still receives too little attention. Especially the transition from adolescence to adulthood, which is accompanied by a transition from child and adolescent psychiatric clinics to adult psychiatry, is problematic; many patients are lost to follow up at that point. At every age, recommended treatment of ADHD should be multimodal, including psycho-education, pharmacotherapy, and disorder-oriented psychotherapy, including training, cognitive-behavioural therapy, and family or couple therapy if needed (Faraone et al., 2015, Kooij et al., 2010; NICE, 2018).

Pharmacological treatments with the indication for ADHD are traditionally divided into two groups: stimulants and non-stimulants. Methylphenidate and amphetamines are the stimulant options, and atomoxetine, guanfacine, and clonidine are the non-stimulants (Faraone et al., 2015). The same drugs are used across the lifespan in clinical practice, but only methylphenidate, lisdexamfetamine, and atomoxetine are officially approved for treatment of ADHD in childhood *and* adulthood in most European countries (Ramos-Quiroga et al., 2013). Unsurprisingly, there have been many more clinical trials evaluating the efficacy and safety of these drugs in children than in adults (Faraone and Buitelaar, 2010, Fridman et al., 2015). Nevertheless, randomised, placebo-controlled clinical trials and meta-analyses convincingly show the effectiveness and safety of both stimulant and non-stimulant drugs for ADHD also in adults (Epstein et al., 2014, Fridman et al., 2015) (Table 1). The National Institute for Health and Clinical Excellence (NICE) guidelines recommend pharmacotherapy as first-line treatment for adult ADHD (NICE, 2018) and consider methylphenidate as the first choice for the treatment of adults, based on available meta-analytical evidence. Apparent differences in the effect size for methylphenidate across the lifespan are likely due to different dosing regimens applied in the clinical trials. Doses around 1 mg/kg of methylphenidate are correlated with better efficacy, yet are rarely achieved in studies of adult patients. Regarding amphetamines or atomoxetine, the situation is similar; highest doses are related with best efficacy. Another factor that may explain the discrepancies in effect sizes is the presence of comorbid disorders. As an example, studies that included patients with SUDs have shown comparatively smaller effect sizes. The influence of such comorbidities on treatment outcome is not exclusively observed in pharmacological trials, the same is seen for studies of non-pharmacological treatments.

**Table 1:** Reported effect sizes (standardised mean difference) from meta-analysis for studies of treatment efficacy for ADHD core symptoms in childhood and adulthood.

Treatment and age-group	Treatment type	Effect size	Reference
Childhood: pharmacological treatment	Methylphenidate	0.72	Faraone and Buitelaar (2010)
	Amphetamines	0.99	Faraone and Buitelaar (2010)
	Atomoxetine	0.64	Schwartz and Correll (2014)
	Guanfacine	0.63	Hirota et al. (2014)
	Clonidine	0.44	Hirota et al. (2014)
Childhood: non-pharmacological treatment	Omega-3	0.16	Sonuga-Barke et al. (2013)
	Diets	0.42	Sonuga-Barke et al. (2013)
	Neurofeedback	0.21	Hodgson et al. (2014)
	Multimodal psychosocial	0.09	Hodgson et al. (2014)
	Working memory training	−0.02−0.20	Cortese et al. (2015); Hodgson et al. (2014)
	Behaviour modification	−0.03	Hodgson et al. (2014)
	Parent training	−0.51	Hodgson et al. (2014)
	Self-monitoring	−5.91	Hodgson et al. (2014)
	School-based	−0.26−0.16	Hodgson et al. (2014); Richardson et al. (2015)
Adulthood: pharmacological treatment	Methylphenidate	0.42−0.72	Castells et al., 2011b, Epstein et al., 2014
	Amphetamines	0.72−1.07	Castells et al. (2011a); Fridman et al. (2015)
	Atomoxetine	0.38−0.60	Asherson et al. (2014); Fridman et al. (2015)
Adulthood: non-pharmacological treatment	Cognitive-behavioural therapy	0.43−1.0	Jensen et al. (2016); Knouse et al. (2017); Young et al. (2016)
	Mindfulness-based therapies	0.53−0.66	Cairncross and Miller (2016)

In terms of drug treatment side effects, the most typical are decreased appetite, sleep disturbance, headaches, drowsiness, tearfulness, abdominal discomfort, nausea and vomiting, irritability, mood changes, constipation, fatigue, sedation, and increased blood pressure and pulse. Delays in height and weight are relatively minor with stimulants, often appear to attenuate with time, and seem not to affect ultimate height and weight in adulthood (Fredriksen et al., 2013). The cardiovascular safety of stimulant medications and atomoxetine has been a subject of debate over many years. It is quite surprising that this controversy occurs for drugs such as methylphenidate, which have been on the market for over 50 years. Cohort studies, however, did not find an increase in serious cardiovascular events following ADHD medications in children or adults, although both stimulants and atomoxetine were found associated with slight increases in heart rate and blood pressure (Cooper et al., 2011).

Public concerns that stimulant treatment in childhood and adolescence may increase SUDs in adulthood seem unsubstantiated. A comprehensive meta-analysis of long-term studies indicates comparable outcomes between children with and without medication treatment history for any substance use and abuse or dependence outcome across all substance types (Hamshere et al., 2013); accordingly, Scandinavian registry studies (Chang et al., 2014b, Dalsgaard et al., 2014) found that ADHD medication was not associated with increased rate of substance abuse; if anything, the data suggested a long-term protective effect on substance abuse (Chang et al., 2014b) (see below).

Research of pharmacotherapy in adults with ADHD is still largely lacking, especially with respect to clinical trials comparing different treatments (pharmacological or psychological) head to head as done in a pioneering study (Philipsen et al., 2015). At the same time, it is necessary to improve the external validity of these studies. Most of the existing clinical trials in this area are the ones performed to reach indication for adults for a particular drug. These studies used very restrictive inclusion and exclusion criteria, rarely reflecting real-life patients. A second area, in which more research is needed, is the systematic assessment of the efficacy and safety of ADHD treatment in the presence of comorbid disorders, especially psychosis. Finally, it is known that around 30% of ADHD patients do not respond to currently available treatments (Faraone et al., 2015). It is therefore important to investigate new pharmacological targets beyond the dopaminergic and noradrenergic systems, preferably including new knowledge on the biological pathways involved in ADHD aetiology (see below). One line of evidence comes from a trial of low dose cannabinoids, which found moderate to large effects on core ADHD symptoms in the absence of adverse effects; this study implicates the cannabinoid system as a potential new target for drug development (Cooper et al., 2017).

It is important to highlight the positive impact of ADHD treatment on aspects of daily functioning. Although initial studies were disappointing regarding the longer-term effects of treatment (Molina et al., 2009), the studies from Scandinavian registries impressively document positive outcomes of treatment, as discussed below.

In addition to pharmacotherapy, several non-pharmacological approaches are used in the control and management of ADHD across the lifespan (Table 1). In many countries, for children and adolescents with mild ADHD, non-pharmacological interventions are the first-line treatment. In moderate or severe cases, the recommendation is to combine non-pharmacological treatment and drug treatment (Faraone et al., 2015). Thus far, non-pharmacological treatments in childhood have demonstrated lesser efficacy in reducing ADHD core symptoms than ADHD drugs (Sonuga-Barke et al., 2013), but may have important benefits for co-occurring comorbidities and behavioural problems. In fact, only free fatty acid supplementation and exclusion of artificial food colour from the diet demonstrated significant beneficial effects on ADHD core symptoms during childhood in a rigorous meta-analysis, although the effects were relatively small (Chronis et al., 2006; Nutt et al., 2007, Philipsen, 2012, Sonuga-Barke et al., 2013, Young et al., 2015). Although cognitive-behavioural therapy (CBT) approaches have not been proven efficacious in children (Sonuga-Barke et al., 2013), the first studies have reported that individual CBT (Antshel et al., 2014) and group CBT (Vidal et al., 2015) could be effective treatments for adolescents with ADHD.

Of particular importance is the transition from childhood to adult services, requiring continuation of both medical and psychological support (Nutt et al., 2007). As indicated above, for adults, pharmacological treatment is the recommended first choice. Group CBT has been proven to benefit adults with ADHD (Solanto et al., 2010), but one study showed that highly structured group intervention did not outperform individual clinical management with unstructured support with regard to the core ADHD symptoms (Philipsen et al., 2015). Promising findings also raise the possibility of mindfulness-based interventions as an effective treatment for ADHD symptoms (Cairncross and Miller, 2016, Hepark et al., 2015). For other types of non-pharmacological treatments, such as neurofeedback, more evidence for efficacy from randomised clinical trials with blinded assessment is necessary for both adolescents and adults (Cortese et al., 2016).

## 6 Disease outcome with and without treatment

Assessment of disease outcome in ADHD requires longitudinal study designs, which are generally scarce. As a result, also the knowledge about the role of treatment during the different phases of life in such outcomes is still patchy. Some clinically-based studies of high quality that have followed up pre-adolescents with ADHD into adulthood are already available and have been instrumental in documenting the disease outcomes of ADHD in different aspects of life. Those include academic, occupational, and social aspects as well as morbidity and mortality. It has been shown that ADHD is associated with academic outcomes, such as poor academic performance (e.g., lower grade point average and increased rates of grade retention) (Galera et al., 2009) and lower rates of high-school graduation and post-secondary education (Galera et al., 2009, Klein et al., 2012, Mannuzza et al., 1993). ADHD is also associated with negative occupational outcomes such as unemployment (Biederman et al., 2006, Klein et al., 2012), having trouble keeping jobs (Barkley et al., 2006, Biederman et al., 2006), financial problems (Barkley et al., 2006, Klein et al., 2012), and work incapacity in terms of sickness absence (Kleinman et al., 2009, Secnik et al., 2005). Those studies have also shown that individuals with ADHD are at increased risk for poor social outcomes such as high rates of separation and divorce (Biederman et al., 2006, Klein et al., 2012), residential moves (Barkley et al., 2006), and early parenthood (Barkley et al., 2006).

In terms of mortality, several studies from Scandinavia have explored the association of ADHD with mortality and which factors are likely to increase mortality. These studies have used information from national registers in Sweden and Denmark that have been linked using a unique person identifier. A Danish register-based study has found that ADHD is associated with significantly increased mortality rates, and that the excess mortality in ADHD is mainly driven by deaths from unnatural causes, especially accidents (Dalsgaard et al., 2015b). Other register-based studies have confirmed that individuals with ADHD are at increased risk for serious transport accidents (Chang et al., 2014a), and also show increases in criminality (Dalsgaard et al., 2013, Lichtenstein et al., 2012) and suicidal behaviour (Ljung et al., 2014), which are severe negative outcomes in their own right and could contribute to the increased mortality.

Systematic reviews of the long-term disease outcomes of treated versus untreated ADHD are somewhat inconsistent. This is partly because long-term observational studies are often limited by a lack of data about treatment compliance and can be confounded by indication, since more severe cases will be more likely to be treated. A systematic review of randomised controlled trial open-label extension studies and naturalistic studies of adults with ADHD concluded that ADHD medications have long-term beneficial effects and are well tolerated, but that more longitudinal studies of long duration need to be performed (Fredriksen et al., 2013). A second systematic review of both childhood and adult studies found that ADHD individuals left untreated had poorer long-term outcomes compared to treated individuals in several major categories including academic, antisocial behaviour, driving, non-medicinal drug use/addictive behaviour, obesity, occupation, services use, self-esteem, and social function outcomes, but that treatment did not result in normalisation (Shaw et al., 2012). In contrast, a third systematic review of placebo-controlled discontinuation studies and prospective long-term observational studies concluded that ADHD medication reduced ADHD symptoms and impairments, but that there was limited and inconsistent evidence for long-term medication effects on improved social functioning, academic achievement, employment status, and psychiatric comorbidity (van de Loo-Neus et al., 2011). Long-term placebo controlled trials would be needed to allow definite conclusions, yet those are almost impossible to conduct in real life.

Pharmaco-epidemiological analyses of large-scale databases, such as the national registers in Scandinavia, are an alternative source of information about potential (long-term) effects of ADHD medication on important disease outcomes. A large register-based Swedish study of adults with ADHD found that treatment with ADHD medication significantly reduces the risk for criminality (Lichtenstein et al., 2012). Another Swedish register-based study found that adult males with ADHD had a 58% reduced risk of serious traffic accidents in periods receiving treatment, compared with periods without treatment (Chang et al., 2014a). This was confirmed in a recent study based on individuals with ADHD from a large insurance claims database, which found similar results in a US setting and also among females (Chang et al., 2017). Three additional pharmaco-epidemiological studies in children and adolescents, using data from a Danish record linkage of national registers (Dalsgaard et al., 2015a), a Hong Kong electronic medical records database (Man et al., 2015), and a German insurance database (Mikolajczyk et al., 2015), have further extended such findings to these age groups. A Danish register-based study showed that treatment with ADHD medication reduced the risk for injuries by up to 43% and emergency ward visits by up to 45% in children with ADHD (Dalsgaard et al., 2015a).

The pharmaco-epidemiological studies can also help clarify the potential role of medication in causing morbidity. For example, two register-based studies of adolescents and adults with ADHD suggest that the co-occurrence of ADHD and suicidal behaviour is due to shared familial risk factors (Ljung et al., 2014), rather than to harmful effects of ADHD medications (Chen et al., 2014). A recent study in a self-controlled case series also suggests that the observed elevation of suicide attempt risk after medication initiation is not causally related to the effects of stimulants (Man et al., 2017). In this study, the incidence of suicide attempt was higher in the period immediately before the start of stimulant treatment. The risk remained elevated immediately after the start of stimulant treatment and returned to baseline levels during continuation of stimulant treatment. Furthermore, the apparent increase in SUD risk in patients treated with stimulants, as discussed above, seems to be due to familiality rather than medication effects. The previous concerns that stimulants could lead to an increase in SUDs could not be confirmed by the pharmaco-epidemiological studies; these studies rather suggest a reduction in SUDs following medical treatment of ADHD (Chang et al., 2014b, Skoglund et al., 2015). Another register-based study from Sweden suggests that ADHD medication does not increase the risk of later depression; also in this case, medication was associated with a reduced risk for subsequent and concurrent depression (Chang et al., 2016).

## 7 Changes in cognitive and neuroimaging profiles across the lifespan

In addition to a lifespan perspective on phenotypic and outcome parameters in ADHD, also the dynamics across life in biological markers and risk factors for the disorder need to be understood better. ADHD is associated with several cognitive impairments and brain alterations, both in childhood and in adulthood. Cognitive deficits in ADHD encompass both higher-level, effortful cognitive functions (e.g., inhibitory control, visuo-spatial and verbal working memory, sustained attention) and lower-level, potentially more automatic cognitive processes (e.g., temporal information processing and timing, vigilance, intra-individual variability, reward processing) (Karalunas et al., 2014, van Lieshout et al., 2013, Willcutt et al., 2005). Meta-analyses of cognitive studies in children establish ADHD to be associated with poorer performance on tasks measuring inhibition, working memory, planning, and vigilance (Huang-Pollock et al., 2012, Willcutt et al., 2005). ADHD is also associated with lower IQ scores (Frazier et al., 2004). In addition, more recent studies, including meta-analyses, indicate a strong association of ADHD with reaction time variability (RTV), capturing lapses in attention (Frazier-Wood et al., 2012, Karalunas et al., 2014, Kuntsi et al., 2010). Studies of adults with ADHD reveal overall similar patterns of cognitive impairments as found in children and adolescents (Coghill et al., 2014, Frazier-Wood et al., 2012, Hervey et al., 2004, Kuntsi et al., 2010, Mostert et al., 2015, Mowinckel et al., 2015, Sonuga-Barke et al., 2010).

Structural and functional neuroimaging studies have documented abnormalities in brain anatomy and function in individuals with ADHD (Cortese et al., 2012, Frodl and Skokauskas, 2012, Greven et al., 2015). Meta-analyses of magnetic resonance imaging (MRI) studies show smaller volumes in the ADHD brain, most consistently in the basal ganglia (Frodl and Skokauskas, 2012, Hoogman et al., 2017). This is important considering the key role of basal ganglia in cognitive deficits typically observed in ADHD, like reward processing. In particular, smaller globus pallidus, putamen, caudate nucleus, nucleus accumbens, amygdala, and hippocampus, but also smaller total intracranial volume, were found in children with ADHD, while in adults with ADHD, the subcortical volume reductions were less pronounced (Hoogman et al., 2017). A smaller anterior cingulate cortex volume had been found earlier (Frodl and Skokauskas, 2012). One relatively large study further reported reductions in total brain and total grey matter volume in children, adolescents, and adults with ADHD, but no alterations in white matter volumes (Greven et al., 2015) (but also see Onnink et al., 2015). Functional abnormalities are documented by a meta-analysis of 55 task-based functional MRI (fMRI) studies (Cortese et al., 2012), reporting that children with ADHD show a hypoactivation in the fronto-parietal and ventral attentional networks involved in executive function and attention, and a hyperactivation in the sensorimotor network and default-mode network (DMN), involved in lower-level cognitive processes. Adult ADHD is, instead, mostly associated with a hypoactivation in the fronto-parietal system and a hyperactivation in the visual network, dorsal attention network, and DMN (Cortese et al., 2012). Atypical brain activity has further been reported using EEG. For example, children and adults with ADHD show alterations in event-related potential (ERP) activity of attentional allocation, inhibition, preparation and error processing during cognitive tasks (Albrecht et al., 2013, Cheung et al., 2016, Geburek et al., 2013), and in quantitative EEG frequency power, mostly increased power of low frequency activity, during resting state (Kitsune et al., 2015).

Overall, cross-sectional studies indicate that many cognitive and brain abnormalities are associated with ADHD in both children and adults, although some differences have also been observed between age groups. Looking at the developmental trajectories of cognitive, neuroanatomical and neurofunctional alterations in ADHD across the lifespan, it is important to examine whether cognitive and brain abnormalities (1) show an age-independent, consistent association with ADHD across the lifespan; (2) are predictors in childhood of later ADHD outcome; (3) can differentiate between individuals with persistent ADHD (from here on “ADHD persisters”) and individuals who have remitted from ADHD over time (from here on “ADHD remitters”).

As for point (1), prospective longitudinal studies with repeated assessments of ADHD and cognitive measures are needed to confirm cross-sectional studies suggestive of similar impairments across the lifespan. Such studies to date, mostly focusing on higher-level cognitive functions using IQ tests, executive functioning and attentional tasks, show that impairments tend to persist from childhood to adolescence and early adulthood in ADHD persisters (Biederman et al., 2009, Cheung et al., 2016, Hinshaw et al., 2007, McAuley et al., 2014). Fewer prospective longitudinal studies have investigated the developmental association between ADHD and lower-level cognitive impairments, such as intra-individual variability measured with RTV. The three largest studies conducted to date on RTV, using motor timing and attentional paradigms, indicate persisting impairments both from middle to late childhood (Vaughn et al., 2011) and from childhood to adolescence/early adulthood in ADHD persisters (Cheung et al., 2016, Thissen et al., 2014). However, in one of these studies the RTV impairment during a motor timing task did not persist in the oldest group of adults investigated, aged 22 years and above (Thissen et al., 2014). Other longitudinal studies have used smaller samples (Doehnert et al., 2010, Doehnert et al., 2013), not distinguished between persisters and remitters (Doehnert et al., 2013, Moffitt et al., 2015), or used different control groups in childhood and at follow up (McAuley et al., 2014), making comparability to the above findings less clear. These studies showed that deficits in visual processing, vigilance, inhibition, and IQ may continue in adult age (Moffitt et al., 2015), while continuity of RTV impairments were observed from childhood to adulthood (Doehnert et al., 2013), but not in adolescence (Doehnert et al., 2010, McAuley et al., 2014).

Among neuroimaging studies, longitudinal studies using multiple assessments from childhood to adulthood are scarce, and have mainly examined neuroanatomical abnormalities with MRI. Available MRI studies consistently show that smaller brain volumes and morphometric abnormalities persist over time in ADHD persisters re-assessed in adolescence and early adulthood (Castellanos et al., 2002, Shaw et al., 2006, Shaw et al., 2013). For example, developmental abnormalities in cortical thinning have been observed in the medial and dorsolateral prefrontal cortex (components of networks supporting attention, cognitive control, and the DMN), which resulted in a pattern of non-progressive deficit in persistent ADHD from childhood to young adulthood (Shaw et al., 2013). Cortical thinning has also been found in ADHD in the medial and superior prefrontal and precentral regions, especially for ADHD individuals with worse clinical outcome: the ADHD group followed a similar developmental trajectory to control individuals from initial assessments in childhood to final assessments in adolescence, but with reductions of cortical thickness at all assessments (Shaw et al., 2006). Atypical developmental patterns of brain anatomy in individuals with persistent ADHD have been reported for subcortical regions and the cerebellum (Castellanos et al., 2002, Mackie et al., 2007, Shaw et al., 2014). Another study found divergent developmental trajectories from age 4 through 19 years for ADHD persisters and control individuals in the basal ganglia from childhood to adulthood: the ADHD group showed a smaller surface area in childhood and a progressive atypical contraction compared to the control group, which instead showed an expansion with age (Shaw et al., 2014). A similar developmental trajectory was found for cerebellar volume, where individuals with persistent ADHD showed decreases in volume from childhood to adolescence/young adulthood, which were not observed in control individuals (Mackie et al., 2007). Further MRI studies have examined the development of the corpus callosum and cortical surface area and gyrification (Gilliam et al., 2011, Shaw et al., 2011), but without distinguishing between persistent and remittent ADHD at follow up, which makes the findings less informative. Little data exist on the continuity of functional brain alterations in ADHD. A small-scale longitudinal EEG study (n = 11 young adults with ADHD) reported that, among ERP impairments related to attentional allocation, inhibition, and response preparation observed in childhood during a cued continuous performance test (CPT), only deficits in response preparation were associated with ADHD in adulthood (Doehnert et al., 2013), without differentiating between persistent and remittent ADHD at follow up.

The prediction of future ADHD outcome (remission/persistence) based on early cognitive and neural impairments measured in childhood *within* an ADHD sample (point 2) is important for early identification of those at risk for worse long-term outcomes (van Lieshout et al., 2013), with an eye on optimising treatment. Studies examining such prediction at short term indicate that impairments in early childhood in executive functions, especially inhibition and working memory, and in IQ predict ADHD symptoms in later childhood (Berlin et al., 2003, Brocki et al., 2007, Campbell and von Stauffenberg, 2009, Kalff et al., 2002), whereas RTV during a CPT does not (Vaughn et al., 2011). Studies investigating clinical outcomes of ADHD persistence/remission with follow up in adolescence and adulthood have obtained inconsistent results. More recent follow-up studies of children with ADHD suggest that RTV and working memory across different tasks in childhood may predict ADHD symptoms and/or functional impairment in adolescents and young adults, even when controlling for childhood ADHD symptoms (Sjowall et al., 2015, van Lieshout et al., 2016a). This is inconsistent with results of studies examining persistence/remission of ADHD as later outcome, which found no evidence for association of aggregated measures of executive function, sustained attention, inhibition, working memory, and RTV in childhood and ADHD persistence/remission in adolescence and adulthood (Biederman et al., 2009, Cheung et al., 2015b, Mick et al., 2011). In a follow-up study, IQ was the only cognitive measure in childhood which predicted later ADHD persistence/remission, while measures of working memory (digit span backward), sustained attention (omission errors), inhibition (commission errors) and RTV from reaction-time and go/no-go tasks did not (Cheung et al., 2015b). The predictive value of IQ has been replicated in two other samples of young adults (Agnew-Blais et al., 2016, Gao et al., 2015), but not in a third sample (Francx et al., 2015b). The only study to date to examine the predictive value of childhood brain activity on adult ADHD outcome indicated that resting-state EEG measures in the theta and beta bands predict ADHD persistence/remission (Clarke et al., 2011), especially in frontal regions implicated in ADHD.

As for point (3), the identification of cognitive and neural processes underlying the trajectories of persistence and recovery from childhood-onset ADHD during the transition to adulthood may further contribute to the prevention of negative long-term outcomes. It has been hypothesised that the persistence of ADHD would be predicted by the degree of maturation and improvement over time in higher-level cognitive function, and lower-level cognitive functions would be linked to the presence of ADHD in childhood irrespective of later clinical status (Halperin and Schulz, 2006). In a follow-up study of almost 100 individuals with childhood ADHD assessed with both cognitive performance and EEG actigraph measures (mean age at follow up 18.30, SD 1.60), ADHD remitters did not differ from controls in higher-level cognitive functions (e.g. working memory and commission errors), but were still impaired in measures associated with lower-level cognitive processes (RTV and perceptual sensitivity) and ankle movement level (Halperin et al., 2008). The latter finding was supported by a second study in the same sample, where RTV did not distinguish ADHD remitters from persisters, both of whom were impaired compared to controls (Bedard et al., 2010). Other studies, however, have not found an association between ADHD remission and improvements in executive functioning (Biederman et al., 2009), interference control (Pazvantoglu et al., 2012), and response inhibition (McAuley et al., 2014). Working memory impairments in young adults diagnosed with ADHD in adolescence compared to controls have also been observed regardless of whether they still met an ADHD diagnosis (Roman-Urrestarazu et al., 2016). Further studies found, across different cognitive tasks, that cognitive- EEG measures of preparation, intra-individual variability (RTV), vigilance, and error processing (mostly reflecting lower-level cognitive functions) differentiated ADHD remitters from persisters assessed in adolescence and young adulthood (Cheung et al., 2016, James et al., 2017, Michelini et al., 2016a). Cognitive and brain activity measures of executive control of inhibition, working memory, and conflict monitoring (largely reflecting higher-level cognitive functions) were not sensitive to persistence/remission of the disorder (Cheung et al., 2016, Michelini et al., 2016a). As such, these studies may suggest that (“lower-level”) preparation-vigilance – instead of higher-level – cognitive functions may be markers of ADHD recovery, following the symptom level at follow up.

Large-scale neuroanatomical studies also found evidence of differences between ADHD persisters and remitters in adolescents and adults in measures of brain volume and structural connectivity. ADHD remitters have shown a slower rate of cortical thinning from childhood to adulthood compared to persisters, such that brain dimensions may be more similar to those of control individuals in frontal and parietal regions with age (Shaw et al., 2006, Shaw et al., 2013). Structural connectivity impairments in the left corticospinal tract, implicated in the control of voluntary movements, have been reported in adolescents and young adults with childhood ADHD with persistent hyperactive-impulsive symptoms compared to individuals with greater symptom improvements over time and control individuals (Francx et al., 2015b). In white matter tracts connecting various regions related to sensorimotor and higher-level cognitive functions, however, both ADHD persisters and remitters showed impairments in adulthood compared to controls (Cortese et al., 2013). Developmental pathways of brain functioning may also show potential abnormalities, as suggested by studies of adolescents with a diagnosis of ADHD in childhood, but limited evidence is available to date. Two studies of adolescents and young adults reported increased resting-state fMRI connectivity in ADHD remitters compared to controls in the executive control network, with intermediate connectivity profiles in persisters (Francx et al., 2015a), and increased EEG connectivity during an executive control task in both ADHD remitters and persisters (Michelini et al., 2017). Other small-scale fMRI studies further suggest that thalamic and cortical activation during response preparation (Clerkin et al., 2013) and caudate activation during working memory performance (Roman-Urrestarazu et al., 2016) may be reduced in both ADHD persisters and remitters. Instead, lower functional correlation between posterior cingulate and medial prefrontal cortices (major components of the DMN) during rest (Mattfeld et al., 2014), lower connectivity between the thalamus and prefrontal regions during response preparation (Clerkin et al., 2013), and lower activations in areas of the prefrontal cortex involved in reward processing (Wetterling et al., 2015) may distinguish ADHD persisters from remitters and controls.

Overall, despite some inconsistencies between studies, some convergence for cognitive and neuroimaging markers of ADHD persistence and remission is starting to emerge. For example, the majority of studies to date show that impairments in executive function do not distinguish ADHD remitters and persisters (Biederman et al., 2009, Cheung et al., 2016, McAuley et al., 2014; Michelini et al., 2016a, 2017; Pazvantoglu et al., 2012). A particularly critical issue, likely explaining some of the discrepancies across studies, is variability in the way the persistence and remission are defined, as studies differ in the use of parent- or self-reports and on whether functional impairment is taken into account at follow-up assessments. However, there is a relatively low agreement between self- and parent-reports of ADHD in adolescents and young adults, and objective cognitive and neurophysiological data show lower agreement with ADHD outcome in adolescence and young adulthood based on self-report than on parent-report (Du Rietz et al., 2016).

## 8 The role of genetic and environmental risk factors and their interplay in ADHD across the lifespan

While investigations into the dynamics of cognitive and neuroimaging markers across life have gained traction, the research into lifespan aspects of underlying risk factors for ADHD is still very much in its infancy. ADHD has a strong genetic component. Family studies have consistently shown familial clustering, with an ADHD relative risk of about 5- to 10-fold in first-degree relatives of probands with ADHD (Biederman, 2005, Biederman et al., 1990, Franke et al., 2012). Twin studies show heritability estimates between 70% and 80%, and the underlying genetic architecture of ADHD appears similar across the different core symptom dimensions and gender (Faraone et al., 2005, Larsson et al., 2014, Nikolas and Burt, 2010). Consistent evidence supports stability in the ADHD heritability across the lifespan estimated using the same informant across ages and cross-informant approaches (Brikell et al., 2015, Chang et al., 2013, Kuntsi et al., 2005).

Given the multifactorial, polygenic nature of ADHD, genetic research has mainly focused on common variants through hypothesis-driven candidate gene association studies (CGAS) and genome-wide association studies (GWAS) with case-control or family-based designs. These designs consist on observational studies in which the frequencies of specific common genetic variants within candidate genes for ADHD or a genome-wide set of polymorphisms are compared between affected (cases) and unaffected (controls) individuals or between affected subjects and their relatives. Several linkage studies have also been performed. They allow genetic mapping of complex traits showing familiar aggregation by assessing the co-segregation of the disease phenotype with sequence variants across the genome. More recently, genetic studies in ADHD have also focused on rare variation (allele frequency <0.05) through copy-number variant (CNV) analyses, which inspect alterations in genomic segments of more than 1Kb in length, and exome chip analysis and whole-exome sequencing, targeting point mutations or small indels. These investigations have shown converging evidence for common biological pathways underlying ADHD, which highlights that both common and rare genetic variants account for a significant proportion of the genetic susceptibility to the disorder (Martin et al., 2015b, Stergiakouli et al., 2012, Zayats et al., 2016).

To date, seven genome-wide linkage scans have been performed in ADHD. Although very little overlap was observed between analyses, different genetic loci potentially involved in ADHD have been found on chromosomes 5p13, 14q12, and 17p11, and a meta-analysis of ADHD linkage studies confirmed a locus on chromosome 16 (Zhou et al., 2008). Only few positional candidate genes have, however, been identified from these linkage scans, e.g. *DIRAS2* (Reif et al., 2011) and *LPHN3* (Arcos-Burgos et al., 2010). Also, although hundreds of CGAS have been reported, only a few findings have been consistently replicated across studies. These studies have focused primarily on genes involved in neurotransmission, particularly in the monoaminergic pathways. Serotonin and dopamine receptors and transporters are the most extensively studied and replicated across populations (Franke et al., 2012, Gizer et al., 2009, Hawi et al., 2015). Almost all studies were performed in children, and none of the resulting candidate genes can be considered as established. One of the few genes tested thoroughly in both children and adults is the dopamine transporter, and intriguingly, opposite alleles were associated with childhood and adulthood ADHD (Franke et al., 2010).

GWAS on ADHD have been completed in nine independent datasets (Anney et al., 2008, Franke et al., 2009, Hinney et al., 2011, Lasky-Su et al., 2008, Mick et al., 2010, Neale et al., 2008, Sanchez-Mora et al., 2015, Stergiakouli et al., 2012, Yang et al., 2013, Zayats et al., 2015), three of them focusing on the persistent form of the disorder (Lesch et al., 2008, Sanchez-Mora et al., 2015, Zayats et al., 2015). Although none of them, nor two meta-analyses on several of these datasets (Neale et al., 2010, van Hulzen et al., 2016), reported genome-wide significance, the integration of top findings from the different studies showed enrichment of genes related to neurobiological functions potentially relevant to ADHD, such as neurite outgrowth, central nervous system development, neuronal development, differentiation and activity, neuron migration, synaptic transmission, axon guidance, Calcium-activated K^+^ channels, FGFR ligand binding, and activation or potassium channels, among others (Mooney et al., 2016, Sanchez-Mora et al., 2015, Yang et al., 2013, Zayats et al., 2015). For the first time, a very recent GWAS meta-analysis in 20,183 ADHD cases and 35,191 controls, including children and adults from 12 datasets, reported genome-wide significant hits in 12 independent loci that include genes involved in neurodevelopmental processes, such as *FOXP2 or DUSP6,* and evolutionarily conserved genomic regions (Demontis et al., 2017). Limited overlap exists between results of GWAS and previous CGAS or linkage studies, and a separate analysis for the persisting versus remitting forms of ADHD is still lacking. Thus, the genetic architecture underlying the lifespan trajectory of ADHD is still largely obscure.

Although each of the ADHD-associated variants appears to account for a small proportion of the variance in ADHD symptoms, SNPs were estimated to account for 10% to 28% of the heritability of the disorder (Anttila et al., 2018, Demontis et al., 2017, Lee et al., 2013a). These studies also highlight substantial genetic overlap between the ADHD genetic background and ADHD-related traits, other psychiatric and neurological disorders or behavioural-cognitive traits, including positive correlations with major depressive disorder, migraine, obesity, and smoking, as well as negative correlation with educational outcomes and childhood IQ (Anttila et al., 2018, Demontis et al., 2017, Lee et al., 2013a). In addition to GWAS, under the hypothesis that ADHD can be explained by the ensemble of genetic markers of small effect, polygenic risk score approaches have emerged to assess whether, when considered “en masse”, ADHD common risk variants also contribute to different ADHD-related phenotypes. These findings provided evidence that polygenic risk for ADHD predicts hyperactivity-impulsivity and inattention traits in the general population, as well as autism spectrum disorder-related traits and conduct disorder (Hamshere et al., 2013, Martin et al., 2014). Again, none of these studies considered the lifespan perspective.

Research on rare variants involved in ADHD points at a greater burden of large CNVs (> 100 kb or > 500 kb) in children or adolescents with the disorder (Martin et al., 2015b, Stergiakouli et al., 2012, Williams et al., 2010) and in adults with ADHD (Lesch et al., 2011, Ramos-Quiroga et al., 2014), and also shows enrichment of ADHD-related CNVs at loci previously associated with neurodevelopmental disorders, such as autism and schizophrenia (Thapar et al., 2016). Additional evidence from exome sequencing supports the involvement of rare variants (minor allele frequency (MAF) of <1%) in individuals with ADHD at different age ranges. Although the study of a pedigree with several affected individuals failed to identify causal rare variants for ADHD (Lyon et al., 2011), rare de novo missense variants were found in brain-expressed genes in children with sporadic ADHD (Kim et al., 2017) and novel putative functional rare variants in the *BDNF* were identified in children and adolescents with ADHD (Hawi et al., 2017). Rare missense and disruptive variants were also identified through whole-exome sequencing or exome chip analyses in adult ADHD (Zayats et al., 2016).

Overall, while it is well-established that ADHD is a highly heritable condition with a complex genetic architecture, more research is needed to identify the specific genetic underpinnings of the disorder and its persistence into adulthood. In addition to risk factors stably involved in ADHD throughout the lifespan, the susceptibility to ADHD persistence may be a dynamic process, with specific genetic influences acting at different developmental stages (Chang et al., 2013, Kuntsi et al., 2005). Thus, different sets of genes, and even different alleles at a given risk locus, could be involved in the differentiation between persisting and remitting forms (Franke et al., 2012, Kuntsi et al., 2005) and influence the disorder and associated cognitive deficits differently according to age (gene by age interactions) (Thissen et al., 2015). To our knowledge, all genetic studies that have approached the problem of the potential differential genetic load of ADHD in persisting versus remitting cases so far have simply compared children and adults with ADHD. Although these reports describe genetic specificities in age groups (Franke et al., 2012, Ribases et al., 2009), they may be subject to false-negative findings, as the comparison is between persistent cases and a sample of children likely to be a heterogeneous group of individuals in which the disorder will persist or remit into adulthood. Longitudinal designs constitute an alternative approach that may provide insights into the role of genes in the persistence of ADHD across the lifespan.

## 9 Environmental risk factors and gene-by-environment (GxE) interactions

One explanation for the discrepancy between high heritability estimates for ADHD and the scarcity of replicable gene-disorder associations could be that expression of specific genes is conditional on epigenetic programming. Such programming is influenced by both genetic code and environment of an individual, although the contribution of the environment to the aetiology of the disorder seems to be lower than that of heritable factors (with around 22% of ADHD variance explained by environmental factors (Faraone et al., 2005, Nikolas and Burt, 2010). The concept of gene-by-environment (GxE) interaction is in line with epidemiologic studies revealing – in addition to the genetic risk – associations between ADHD and environmental adversity including pre- and peri-natal risk factors (maternal stress, smoking or alcohol consumption during pregnancy, low birth weight, prematurity), environmental toxins (organophosphates, polychlorinated biphenyls, lead), unfavourable psychosocial conditions (severe early-childhood deprivation, maternal hostility) and nutritional factors (Faraone et al., 2015). It is likely that the heritability estimates for ADHD based on twin research are inflated by GxE interactions (Purcell, 2002), which may also account in part for some inconsistent findings of genetic association. Accordingly, several studies support the influence of genetic variants on effects of environmental risk factors for ADHD (Franke and Buitelaar, 2018), with interactions found e.g. between *DRD4* and exposure to prenatal smoking (Pluess et al., 2009), *SLC6A3/DAT1* and maternal use of alcohol during pregnancy (Brookes et al., 2006), institutional deprivation (Kumsta et al., 2010) psychosocial adversity (Laucht et al., 2007), *SLC6A4*/*5HTT* and psychosocial stress (Muller et al., 2008), *MAOA* and negative parenting behaviour (Li and Lee, 2012), and *ADGRL3*/*LPHN3* and maternal stress during pregnancy (Choudhry et al., 2012).

Most of the environmental factors that are considered to increase ADHD and comorbid disorder susceptibility act prenatally, suggesting that exposure to environmental risks would have more impact if occurring during a critical developmental period (for a review, see Mill and Petronis, 2008, Spiers et al., 2015). Although it should be kept in mind that risk factors associated with a disorder are not necessarily causal (Rutter, 2007), there is now evidence to suggest that early-life exposure to smoking, alcohol, and illicit drug use as well as suboptimal nutrition or the changes in microbiome composition can permanently affect transcriptional regulation through epigenetic alterations, and this is thought to contribute to the long-lasting consequences on offspring health (Loche and Ozanne, 2016). Foetal, perinatal, and adolescent periods are the developmental periods of highest phenotypic plasticity, contributing largely to developmental programming, and the epigenome is sensitive to environmental challenges applied during these critical windows of development. Various risk factors that usually accompany behavioural disorders seem to be involved in these epigenetic modifications, especially early-life stress including trauma, abuse, and neglect. As there is preliminary evidence for a role of diet or lifestyle in ADHD, dietary changes, like the elimination or addition of certain nutrients, are being explored as a possible way to modify symptoms (Lange, 2017). While nutrition and lifestyle, as well as environmental adversity in general, are thought to be potent epigenetic modifiers, complex interactions among food components, environmental toxins, or drugs of abuse and DNA methylation, histone modifications and epigenetic-related RNA-based mechanisms lead to dynamic regulation of gene expression that controls the neural cell phenotype and brain function (Robison and Nestler, 2011). A considerable challenge to research on the human epigenome is, however, the tissue and cell specificity of epigenetic modification. As brain tissue from ADHD patients is not available, research on epigenetic mechanisms has to rely on accessible material (e.g. blood or buccal cells) as a proxy for the brain. Nevertheless, there is evidence that epigenetic research in humans is feasible, and first studies, focusing on both potential risk genes (Heinrich et al., 2017, van Mil et al., 2014) and the entire epigenome (Walton et al., 2017, Wilmot et al., 2016), confirm links between epigenetic modification of genes and ADHD.

A pioneering study on ADHD epigenetics was a whole-epigenome screening of two independent samples in boys with ADHD by Wilmot et al. (2016). It provided evidence for an involvement of gene-sets and pathways related to inflammatory processes as well as modulation of monoamine and cholinergic transmission. Two genes, encoding *VIPR2* and *MYT1L* (mutations cause intellectual disability, autism), were confirmed in the replication sample. Walton et al. (2017) reported that DNA methylation at birth differentiated ADHD-related trajectories in a population-based cohort across multiple genomic locations implicating e.g. genes coding for *SKI* (involved in neural tube development), *ZNF544* (previously linked to ADHD), and *ST3GAL3* (mutations cause intellectual disability), although none of these genes maintained an association with ADHD trajectories at age 7 years. Together, these studies – some in small cohorts – provide initial insight into the epigenetic background of ADHD, underscoring the relevance of differential DNA methylation in genes related to monoaminergic, cholinergic and GABAergic transmitter system function and neurodevelopmental processes that play a role in the formation, maturation, and plasticity of distinct brain networks.

## 10 Discussion and future directions

In this review, we aimed to provide an overview of the current knowledge about ADHD across the lifespan. We covered the major phenotypic and biological issues related to this disorder, which is a prominent cause of psychiatric morbidity and impairment in both childhood and adulthood. We discussed developmental trajectories in core phenotype definition, comorbidity, cognitive and brain structural/functional markers, as well as treatment trajectories, outcomes, and genomic markers. Our review shows that beyond research into the core symptoms of ADHD, information is still very patchy, and relatively little is known about developmental trajectories from childhood into adulthood for most of the issues of interest. This defines priorities in the coming years for research of phenotypic issues, treatment and prediction of outcome, as well as our understanding of the biological underpinnings of ADHD.

In all areas of our investigation into the ADHD literature, the absence of studies in the elderly is highly apparent. Old age is the current blind spot of ADHD research, which has to be factored in when interpreting all the data presented above. This lack of knowledge limits the possibility to perform a review of the complete lifespan, as most available studies deal with ADHD in children – even 50 years after the first studies showed that ADHD also exists in adults and can continue from childhood to adulthood (Wood et al., 1976). Beyond mid-adulthood, hardly any information on ADHD can be found.

An important additional caveat of current literature is the limited availability of longitudinal studies with a careful and repeated, detailed characterisation of participants across different phases of the lifespan regarding terms of deep clinical phenotyping as well as biomarkers. Such work is essential to chart the trajectory of ADHD symptoms over the lifetime and will answer important questions about the interplay of ADHD disposition with environmental conditions. Those studies are urgently needed for clinical cohorts as well as the general population, and should include broad assessment batteries of disease phenotypes and treatments, quantitative behavioural measures, cognitive tasks and neuroimaging, as well as repeated biomaterial collection. Some existing population studies, like MoBA (Magnus et al., 2016), ALSPAC (Martin et al., 2015a), the Dunedin Study (Moffitt et al., 2015), and LifeLines (Stolk et al., 2008) and in particular more recent large-scale initiatives like the UK Biobank (Clarke et al., 2017) may provide a template for such work. Pressing questions for research in such cohorts can be defined in terms of the phenotypic definition of ADHD:

(1) Only in such longitudinal cohorts the relation of apparent adult-onset ADHD to the neurodevelopmental form of the disorder can be defined: are these different disease entities, with different aetiologies and (co)morbidity profiles, or can they be different subtypes/presentations of the same, overarching disorder? To study this question, one needs also to enrol children with mental disorders other than ADHD and sub-threshold ADHD symptoms.
(2) Is the persistent form of ADHD really persistent, or do ADHD symptoms over time fluctuate with varying intensity, thus creating suffering and burden only in specific life phases, e.g. characterised by high life stress or high demands for self-management (Grevet et al., 2006)? Persistent ADHD, after all, might not be as trait-like as it is assumed (Karam et al., 2017), which might obscure its detection.
(3) There are only a few studies on ADHD remitters. However, this particular group brings up important questions: which are the resilience factors that protect against persistence? Are there differential genetic factors that have a role in remission versus persistence? What about environmental factors and GxE phenomena? What is the brain's developmental trajectory? Clues that help to answer these questions might translate into clinical practice to improve the outcome of patients, and thus according studies should be prioritised.
(4) Developmental trajectories of comorbidity profiles and cognitive functioning across the lifespan: as we have reviewed above, ADHD is characterised by extensive comorbidity in childhood and adulthood. However, the comorbidity profiles of child and adult ADHD change and only partly overlap. Clinical longitudinal studies are needed to understand onset and changes in comorbidity. Recent findings from large genetic studies as well as register-based studies show that comorbidity is often based on shared familial and genetic factors (e.g. Ghirardi et al., 2017, Lee et al., 2013a).
(5) Cognitive functioning, brain structure/function, and the contribution of genetic and environmental risk factors across the lifespan: overall, cognitive and neuroimaging studies to date converge in indicating that most impairments persist when ADHD persists from childhood to later assessments. Yet, most existing studies are cross-sectional and have used samples in young or middle adulthood only. Large longitudinal studies are needed, also including assessments of older age groups, to fully characterise the developmental trajectories of ADHD-related cognitive and brain impairments across the lifespan. In addition, since some of the cognitive and neural alterations observed in ADHD are non-specific, but rather shared with other disorders (e.g. autism and bipolar disorder) Rommelse et al., 2011, Michelini et al., 2016b, 2018), future research should take into account comorbid symptoms when examining the persistence of impairments. In terms of genetic and environmental risk factors, we need to clarify the contribution of individual factors across the lifespan. Also, recent work indicates that there is limited specificity in the genetic contribution to ADHD (Demontis et al., 2017) and other psychiatric disorders (e.g. Anttila et al., 2018). In fact, it appears that part of the genetic contribution to psychiatric disorders is due to a general psychopathology factor (or “p-factor”) (e.g. Neumann et al., 2016). How these findings relate to comorbidity and disease outcome in ADHD is another important area of future research.
(6) Finally, prediction of disease outcome is an area of research that can directly influence treatment decisions in daily clinical practice. Cognitive, electrophysiological, and neuroimaging markers associated with childhood ADHD persist into adulthood, but show variable trajectories reflecting both delayed maturation and within-group differences. Findings suggest a pattern of corresponding biomarkers that predict remission versus persistence of ADHD symptoms in adolescence and adulthood. However, to move forward towards potential clinical applications of these markers as a means to identify those childhood and adolescent cases with ADHD that are at high risk for persistent ADHD, prediction parameters at the individual level have to be examined. Importantly, the predictive power/accuracy of currently identified biomarkers is individually low. Future studies should therefore explore whether combining markers from different cognitive and imaging domains as well as (epi)genetic approaches may improve prediction. Future longitudinal and clinical studies should address these questions while using sophisticated approaches, like normative modelling and multivariate pattern recognition (Wolfers et al., 2015). While ADHD outcome is mostly defined as persistence of the clinical diagnosis or symptoms, studying outcomes related to educational performance and adulthood functionality might also be of importance for the quality of life of patients.

The questions raised above reach far beyond mere academic interest, as respective answers will immediately impact on ADHD treatment. For instance, timing of treatment is an essential issue, as we are only about to begin to understand the long-term effects of pharmacological ADHD treatment regarding outcome (educational, socio-economic and, most importantly, health, also regarding comorbid conditions). To address this, however, we need granular longitudinal studies, initiated in childhood and followed up long-term. A tightly linked question is how adolescents can be kept in treatment – or at least in mental health services – during the transitional period, thereby preventing the “transition gap” and potentially the development of comorbid disorders such as depression or SUD. On the other hand, a waxing and waning pattern of ADHD symptoms and related burden of disease in specific life situations might allow for interval treatment, and especially addressing the role of resilience in the interaction of life stress and ADHD disposition might unleash a huge potential for psychotherapy. More research into persistence within and across different age-ranges is therefore urgently needed. Beyond these “traditional” approaches, novel treatments, such as non-invasive brain stimulation, neurofeedback, and nutritional interventions, should be assessed for their lifespan effects. Within this framework of long-term treatment effects, prediction has also to be factored in. If ADHD persistence or development of comorbidity could reliably be foreseen, this would dramatically alter the way ADHD is treated over the lifespan, opening up a perspective for precision medicine. Most likely, predictive approaches will be multimodal (integrating genetics, proteomics, neuroimaging, and granular ecological momentary assessment (EMA) of behavioural patterns), but also require biomarker studies on disease trajectories of the lifespan.

In terms of understanding the risk factors underlying ADHD, which might be linked to such predictive markers, important progress has been made in the areas of environmental and genetic risk factors in recent years. In particular, the latter one has been enabled by technological progress as well as the formation of large, international collaborations, like the Psychiatric Genomics Consortium (Cross-Disorder Group of the Psychiatric Genomics Consortium, 2013). Still, acknowledging the small effect sizes of most individual genetic risk factors for ADHD, much larger samples will be needed in the future to identify a sufficient number of the underlying genes to tease out the most strongly involved (and potentially druggable) biological pathways contributing to ADHD. An excellent development in this area is the increasing availability of biosamples linked to population registers, starting with the Danish iPSYCH Initiative (Demontis et al., 2017). This will likely allow us to reach sample sizes of over 100,000 patients in the next 5 years. In addition, the recent demonstration of the strong genetic overlap of clinically diagnosed ADHD and population ADHD scores (Demontis et al., 2017) will contribute to a strong rise in sample availability and the number of ADHD loci identified in the coming years. Critical windows for environmental risk factors across the lifespan are already understood quite well. However, while we know that partly different sets of genetic factors contribute to ADHD onset and persistence (Chang et al., 2013), the lifespan perspective has yet to be taken in genetics research and is only rarely addressed. The availability of register-data and population samples also increases our prospects of improving our knowledge about differences in the contribution of different genetic risk factors to ADHD across the lifespan.

More than 1000 genes/genomics regions may contribute to neurodevelopmental disorders like ADHD. Many of those genes/regions can be expected to contain several independent risk loci. How informative the identification of all of those will be for our understanding of disease mechanisms and potential new treatment routes has recently been questioned by a paper discussing “omnigenic” diseases and traits (Boyle et al., 2017). In their paper, the authors argue that the most informative genetic markers may come from studies of rare genetic variants, as more or less all genes expressed in a certain tissue can be expected to make small contributions to a disease related to this tissue (Boyle et al., 2017). The role of rare variants in ADHD across the lifespan is currently virtually unexplored (although an exome sequencing study is in the final stages of analysis), and awaits the upcome of affordable genome sequencing approaches.

Lastly, epigenetics research offers great opportunities to integrate genetic and environmental risk in ADHD. Also here, only first steps have been made towards exploring the involvement of aberrant epigenetic patterning in ADHD. As discussed above, these steps are hampered by the tissue specificity of epigenetic modifications, leading to uncertainty about the predictive value of proxy tissues like blood and saliva, which are the only accessible tissues available in large samples. Changes in epigenetic profiles over time, like those needed to explore lifespan changes in the ADHD phenotype/outcome, might be more informative in those proxy tissues, but this hypothesis awaits testing.

In conclusion, ADHD is the prototypic example of a neurodevelopmental disorder starting early in life and developing with a highly variable trajectory. While in some cases it might be the entry point into a negative, burdensome trajectory, other patients may remit and even transform some deficits into adaptive behaviours, living highly successful lives. The latter suggests that there is potential for improvement in many ADHD patients, if recognised early on and receiving optimal treatment. To clarify the course, to identify those at risk of unfavourable outcome and to provide tailored treatment, longitudinal, granular, and multimodal studies are clearly needed. While we appreciate that this poses a challenge, we are confident that such an endeavour would – just like the fight against HIV three decades ago – pay off in every respect to improve the life of many patients.
